# Exposing *Toxoplasma gondii* hiding inside the vacuole: a role for GBPs, autophagy and host cell death

**DOI:** 10.1016/j.mib.2017.10.021

**Published:** 2017-11-12

**Authors:** Jeroen P Saeij, Eva-Maria Frickel

**Affiliations:** 1 School of Veterinary Medicine, Department of Pathology, Microbiology and Immunology, University of California, Davis, Davis, CA 95616, USA; 2 The Francis Crick Institute, Host-*Toxoplasma* Interaction Laboratory, 1 Midland Road, London NW1 1AT, UK

## Abstract

The intracellular parasite *Toxoplasma gondii* resides inside a vacuole, which shields it from the host’s intracellular defense mechanisms. The cytokine interferon gamma (IFNγ) upregulates host cell effector pathways that are able to destroy the vacuole, restrict parasite growth and induce host cell death. Interferon-inducible GTPases such as the Guanylate Binding Proteins (GBPs), autophagy proteins and ubiquitin-driven mechanisms play important roles in *Toxoplasma* control in mice and partly also in humans. The host inflammasome is regulated by GBPs in response to bacterial infection in murine cells and may also respond to *Toxoplasma* infection. Elucidation of murine *Toxoplasma* defense mechanisms are guiding studies on human cells, while inevitably leading to the discovery of human-specific pathways that often function in a cell type-dependent manner.

## Introduction

*Toxoplasma gondii* is an important pathogen of animals and humans with ~30% of the world’s population chronically infected. While immunocompetent people generally control the infection, *Toxoplasma* infection can lead to congenital abnormalities, ocular disease and health problems in the immunocompromised. Although *Toxoplasma* can infect any warm-blooded animal, mice are considered important intermediate hosts as they are natural prey of cats, the definitive host, which is likely why many *Toxoplasma* secreted effectors target murine restriction mechanisms.

*Toxoplasma* can invade any nucleated cell and resides inside the cell in a parasitophorous vacuole (PV). The PV membrane (PVM) shields the parasite from intracellular cytoplasmic defense mechanisms that have evolved to detect cytoplasmic pathogens. However, in both mice and humans, the immune system ultimately controls the initial acute phase of the infection and the parasite transitions into a cyst form that characterizes the chronic state of the infection.

Interferon gamma (IFNγ) is the central cytokine in eliciting anti-*Toxoplasma* effector mechanisms. These mechanisms involve either the direct destruction of the PVM, the acidification of the intravacuolar environment, the starvation of the parasite inside the vacuole or the activation of host cell death upon infection. The parasite restricting strategies ultimately employed depend on the host organism and the cell type (reviewed in [1,2]).

Different *Toxoplasma* strains (e.g. types I, II and III are the classical North American and European strains) vary in their genomes, resulting in divergent resistance to these host defense mechanisms. For example, the polymorphic virulence factors ROP5/ROP18 specifically counteract murine and not human defense mechanisms (reviewed in [3,4,5^••^,^6^^••^] and see Table 1).

By now we have a more detailed picture of how IFNγ-activated mechanisms combat *Toxoplasma* in murine cells, yet we are only at the beginning of understanding human *Toxoplasma* control. At the center of murine *Toxoplasma* control are IFNγ-inducible GTPases, the Immunity Regulated GTPases (IRGs) and Guanylate Binding Proteins (GBPs), regulated by autophagy proteins and interconnecting with ubiquitin-driven pathogen control. Here, we focus on how GBPs, autophagy and ubiquitin restrict the parasite and on how GBPs are linked to host cell death pathways to control infection in general. We elucidate lessons learnt from mouse studies and the current and future roles this knowledge will bring to human studies.

## Divergent cell-autonomous GBP-mediated control of *Toxoplasma* in mice and humans

IFNγ upregulates expression of host IRGs and GBPs. Mice possess 23 IRGs, while humans have only one truncated ubiquitously expressed IRG that is not IFNγ-inducible (IRGM) [7]. IRGM is a risk locus for tuberculosis [8], with a currently unclear molecular function on other intravacuolar pathogens [9]. On the contrary, humans have 7 GBPs, while mice have 11 active GBP family members [7]. IRGs and GBPs collaborate in their function in mice [10,11,12^••^]. Family members of both IRGs and GBPs control *Toxoplasma* through different mechanisms depending on host species and cell type and different *Toxoplasma* strains differ in their susceptibility to IRG/GBP-mediated restriction because of strain differences in effectors that counteract the IRGs/GBPs (see below and Table 1).

## How do GBPs restrict *Toxoplasma* in mice?

**Figure 1:**
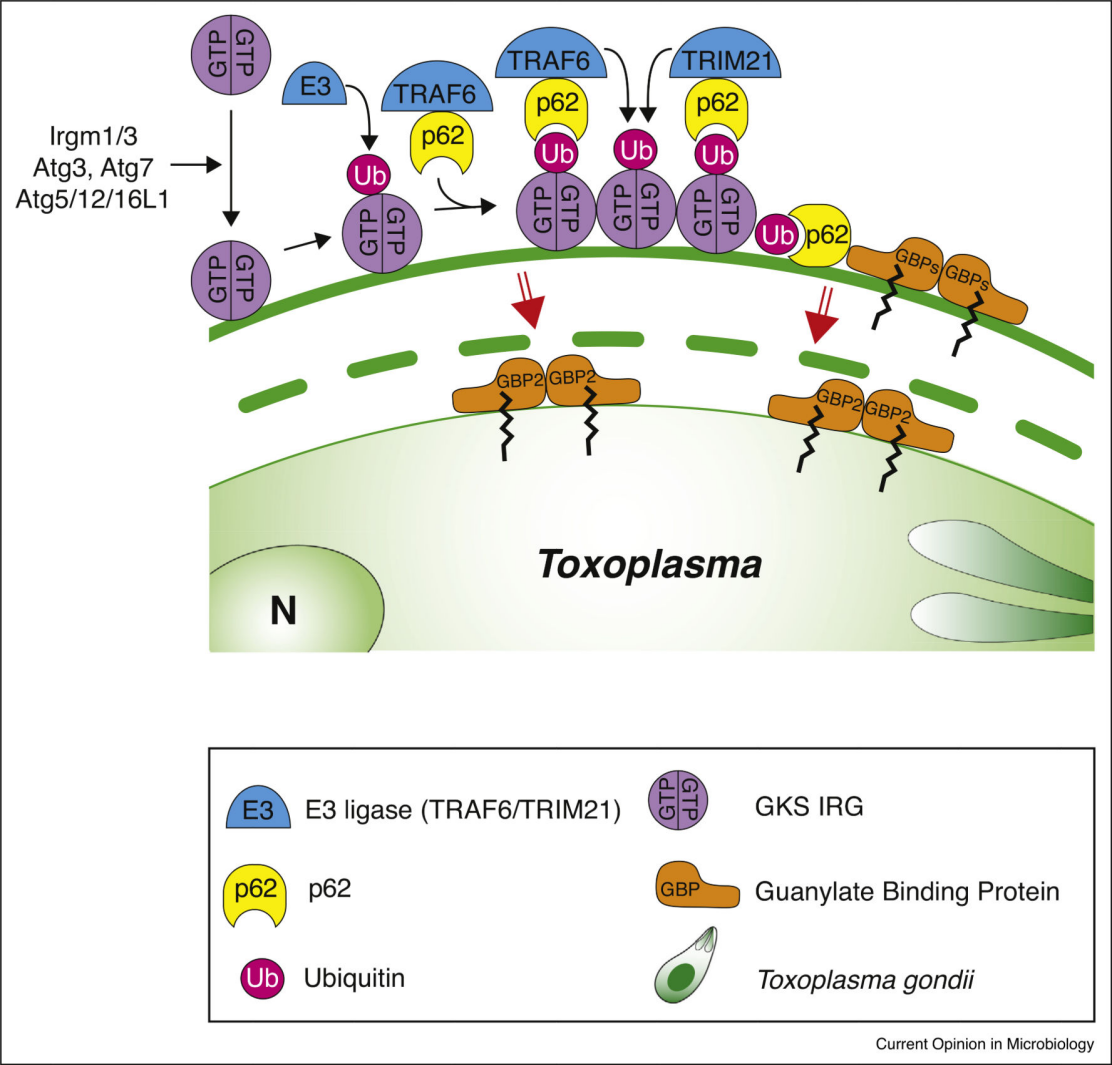
GBP-mediated restriction of *Toxoplasma* in murine cells. In murine cells, type II and III *Toxoplasma* vacuoles are attacked by a range of host proteins leading to the disruption of the vacuolar membrane. The ‘GMS’ IRGs (not shown) block ‘GKS’ IRG activation. Once activated, ‘GKS’ IRGs accumulate on the vacuole and recruit an unknown seeding E3 ubiquitin ligase, as well as the p62-interacting E3 ubiquitin ligases TRAF6 and TRIM21. GBPs target to the vacuole via p62-dependent and independent mechanisms. Ubiquitination of the vacuole is of the K48 and K63 linkage type on substrate proteins that potentially include IRGs and GBPs themselves. Rupture of the vacuole is dependent on IRGs, GBPs and p62.

Three regulatory IRGs are of the ‘GMS’ motif type and keep the effector ‘GKS’ motif type IRGs at endomembranes in an inactive state [13,14]. Upon release, the ‘GKS’ IRGs target pathogen vacuolar membranes devoid of GMS IRGs, enabling a cascade of host defense molecules to accumulate (Figure 1). ROP17/ROP18 in cooperation with ROP5 and GRA7 virulence factors of type I parasites target ‘GKS’ IRGs for phosphorylation keeping the IRGs off the PV (reviewed in [15]), a mechanism that also effects the recruitment of GBPs [16]. Central to the host defense cascade is also ubiquitin, which is deposited onto the vacuole of *Toxoplasma* and *Chlamydia* in a like fashion by a yet unidentified E3 ubiquitin ligase [12^••^]. The scaffold protein p62 binds to ubiquitin and deposits the E3 ubiquitin ligase TNF-receptor associated factor (TRAF)6, which engages in a feedback loop to recruit more ubiquitin [12^••^]. Tripartite Motif Containing (TRIM)21 is another recruited E3 ubiquitin ligase that additionally is critical to *Toxoplasma* defense *in vivo* [17^•^]. Ubiquitin and p62 both enable further recruitment of GBPs to the vacuoles, a pathway that is active in mouse embryonic fibroblasts (MEFs) and murine macrophages [10,11,16,18]. Rab GDP-dissociation Inhibitor (GDI)α acts as a negative regulator of GBP deposition around the vacuole [19]. Both IRGs and GBPs at the PV are essential to disrupt the vacuolar membrane by vesiculation exposing the pathogen inside [10,20]. The exact mechanism of vacuolar disruption is still unknown.

The IRG/GBP GTPases seemingly destroy the vacuoles of only a limited number of vacuolar pathogens: *Toxoplasma gondii*, *Chlamydia trachomatis*, and *Encephalitozoon cuniculi*. *Toxoplasma* and *E. cuniculi* do not enter the host cell through a phagocytic mechanism but have a unique ‘active’ invasion mechanism that excludes most host membrane proteins from the vacuolar membrane [21,22]. However, there are no regulatory IRGs on the host plasma membrane and it is therefore unclear if the specific mode of invasion (e.g. through phagocytosis vs. active invasion) determines why the vacuoles of only some pathogens are targeted by the IRGs [52]. Murine *Chlamydia* species adapted to its specific host avoid IRG/ GBP recognition, but when the human adapted *Chlamydia trachomatis* is for example studied in murine cells it gets targeted by IRGs/GBPs, strongly suggesting putative bacterial inhibitory effectors regulating IRGs [23^•^]. Once effector IRGs bind to the vacuolar membrane and destroy it, the pathogen is exposed and its outer membrane can be targeted for GBP-mediated destruction [24^••^]. Interestingly, the apicomplexan *Plasmodium berghei* in the liver does not get recognized by any member of the IRG family and GKS IRG knockout mice (Irga6) have the same parasite load as infected wild-type mice [25]. Recently it was shown that also the replication complex (RC) of +RNA viruses, a vacuole-like structure these viruses use for their replication, can be targeted by IRGs and GBPs in murine cells and by GBPs in human cells. This IRG/GBP targeting to the RC was necessary for full IFNγ-mediated inhibition of these viruses. Because the membrane of the RC is derived from host endomembranes it is unclear why the regulatory IRGs would not prevent the activation of effector IRGs on the RC. It was proposed that there might be a common unknown PAMP between pathogens targeted by the IRGs/GBPs [26].

## How do GBPs restrict *Toxoplasma* in human cells?

Considerable human cell type variation exists with regard to GBP-mediated restriction of *Toxoplasma*. In HAP1 cells (haploid fibroblast-like leukemia cells) 6% of PVs recruited hGBP1–5 and a total hGBP deletion showed no defect in IFNγ-mediated *Toxoplasma* control [27]. In contrast, hGBP1 is recruited to the PVs of both type I and II *Toxoplasma* and restricts their growth in mesenchymal stromal cells, while hGBP2 and 5 have no functional effect [28]. In epithelial A549 cells, hGBP1 specifically restricts type II *Toxoplasma* replication without targeting the PV [29^•^]. Thus, hGBP recruitment does not necessarily predict its putative defense function in human cells. It remains unstudied whether the PV remained intact in these human cell types and by which mechanism the hGBPs restrict *Toxoplasma*. Of note, hGBP1, 2 and 5 harbor a CAAX box for isoprenylation at their C-terminus, potentially enabling targeting to various membranous compartments, as shown for hGBP1 and the Golgi [30,31]. Furthermore, yet unidentified parasite virulence factors may only interfere with the hGBP system in select human cell types. Currently, no data is available on the GBP-mediated restriction of *Toxoplasma* in human macrophages.

## The role of autophagy in restricting *Toxoplasma* in mice and humans

Autophagy is a catabolic pathway generally used by a cell to clear cytoplasmic material, but it can be extended to the destruction of pathogens [32] (see also the review by I. Coppens in this issue). Importantly, autophagy is tightly regulated by nutrient sensing pathways [33].

## Murine autophagy pathways targeting *Toxoplasma*

**Figure 2:**
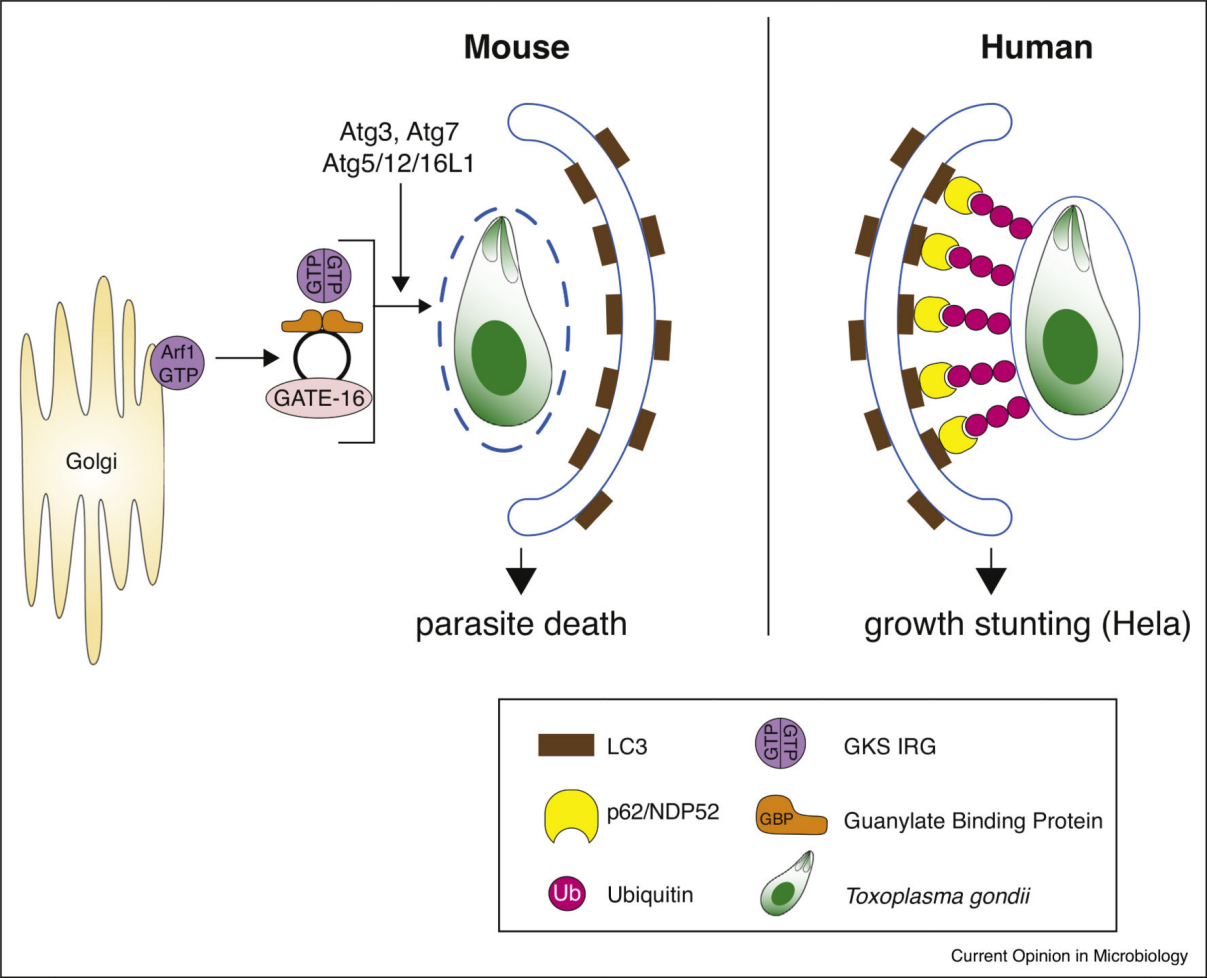
Autophagy-mediated restriction of *Toxoplasma* in human and murine cells. In mice, the Atg proteins Atg7, Atg3 and the Atg12-Atg5-Atg16L1 complex, all involved in delivery and conjugation of LC3 to the autophagosomal membrane, are necessary to target the IRGs and GBPs to the *Toxoplasma* PVM. GATE-16 is the only LC3-like protein essential for controlling *Toxoplasma* infection *in vivo*, by activating the Golgi-localized membrane trafficking regulator Arf1 and keeping GBPs in a non-aggregated form in the cytoplasm of cells. GBPs and IRGs disrupt the PVM and LC3-driven autophagosomes either clear the parasite itself or the membrane remnants that remain. In humans, Atg7/16L1 (not pictured) target ubiquitin to the *Toxoplasma* PVM. This leads to the recruitment of p62 and NDP52 and subsequently LC3, without acidification of the PV and disruption of the PVM. The parasite is eventually enveloped in the autophagic double membrane where it fails to grow and replicate further.

The autophagy-related (Atg) proteins Atg7, Atg3 and the Atg12-Atg5-Atg16L1 complex, which are involved in delivery and conjugation of the ubiquitin-like protein Microtubule-associated protein 1A/1B-light chain 3 (LC3) to the autophagosomal membrane, are necessary to target the IRGs and GBPs to the PVM [27,34–39] (Figure 2). All murine LC3 homologs as well as Gamma-aminobutyric acid receptor-associated proteins (GABARAPs) are also targeted to the PV [35,38]. Park et al. found all LC3 homologues to control IFNγ-driven *Toxoplasma* restriction, while using a different method of analysis for in vitro parasite replication, Sasai et al. deemed only GABARAPL2 (GATE-16) to be essential [38,40^••^]. Regardless, in vivo, only the GABARAPL2 (GATE-16) deficiency rendered mice as highly susceptible to *Toxoplasma* infection as IFNγR^−/−^ mice [40^••^]. This property is by virtue of an ADP-ribosylation factor 1 (Arf1)-binding motif found in GABARAPs, which upon binding to Arf1 possibly activates this Golgi-localized membrane trafficking regulator [40^••^]. Atg3, 5, 7 and 16L1 are additionally needed to deposit ubiquitin and the ubiquitin adaptor protein p62 around the PV, whereby p62 is thought to facilitate MHC class I presentation of vacuolar antigens to CD8 T cells in infected IFNγ-stimulated MEF and DCs [39]. In slight contrast, p62 has been found to directly control *Toxosplasma* in MEFs, via its participation of GBP and TRAF6 recruitment, leading to increased ubiquitin deposition around the PV [37].

It was thought that once the parasite vacuole is destroyed, in a second step, autophagy membranes form around the denuded parasite, clearing it by classical acidification. This process is dependent on Irgm3 [20]. More recently, the notion was put forward that acidic clearance of material involves only the remnant membranes rather than the parasite itself and that canonical degradative autophagy is not required to restrict *Toxoplasma* [27,35,36]. The simple question remains as to what happens to the arguably dying parasite and the material it leaves behind? Possibilities are antigen presentation to CD8 T cells of vacuolar content (reviewed in [41]) and stimulation of host cell death by exposed pathogen material (see below).

## Human autophagy pathways targeting *Toxoplasma*

In IFNγ-stimulated HeLa cells, ubiquitin is deposited around type II and III *Toxoplasma* PVs to mark them for non-canonical autophagy that leads to non-acidic growth stunting [6^••^]. This pathway employs the ubiquitin adaptor proteins p62 and the human-specific Nuclear Domain 10 Protein (NDP)52 and is dependent on ATG7 and ATG16L1. In contrast, in HAP1 cells, ATG16L1 KO does not have an effect on *Toxoplasma* type II restriction and only a marginal effect on GBP recruitment [6^••^] and in human forskin fibroblasts (HFFs) ATG5 knockdown did not impact IFNγ-mediated growth restriction of *Toxoplasma* type I [57]. In human umbilical endothelial vein cells (HUVEC), an autophagy-independent, ubiquitin and p62-dependent endo-lysosomal acidification and elimination of *Toxoplasma* type II was observed [5^••^]. The function of ATG proteins in controlling *Toxoplasma* in human macrophages has not been tested.

Autophagy is a pathway intimately connected with cellular metabolism. In many human cell types IFNγ-mediated restriction of *Toxoplasma* is mediated by the upregulation of Indoleamine-2,3-dioxygenase (IDO), which by degrading L-tryptophan, inhibits the growth of the tryptophan auxotrophic *Toxoplasma* [42]. Surprisingly, GBP versus IDO-mediated restriction of *Toxoplasma* has not been investigated in the same cell type. It is thus possible that these pathways counter-regulate each other and co-exist. For example, nutrient starvation can upregulate autophagy which might redirect proteins important for both autophagy and GBP function (e.g. LC3 and ubiquitin) to autophagosomal membranes instead of the vacuolar membrane. How exactly *Toxoplasma* is restricted in a human cell might depend on the phagocytic ability of the cell versus induced GBP and IDO levels.

## The role of host cell death in restricting *Toxoplasma*-potential roles for GBPs?

GPBs can also mediate a programmed form of host cell death called pyroptosis, which involves the activation of the inactive zymogen Caspase-1 (Cysteine-aspartic protease). Because intracellular pathogens need host cells for replication, destroying this niche is an effective way of inhibiting pathogen growth. Upon recognition of cytoplasmic PAMPs by cytoplasmic PRRs such as the Neuronal Apoptosis Inhibitor proteins (NAIPs), Nucleotide binding Oligomerization (NOD)-like receptors (NLRs) and AIM2-Like receptors (ALRs), macromolecular complexes containing pro-Casp1 get formed upon which Casp1 is activated by proximity-induced autoproteolysis. Active Casp1 then subsequently cleaves the proinflammatory cytokines pro-IL1*β* and pro-IL18 upon which active IL1*β* and IL18 is released. Casp1 also cleaves Gasdermin-D thereby removing the N-terminus-mediated inhibition of the C-terminal pore-forming domain allowing formation of a multimeric pore in the host cell plasma membrane eventually causing host cell death (reviewed in [43]).

**Figure 3:**
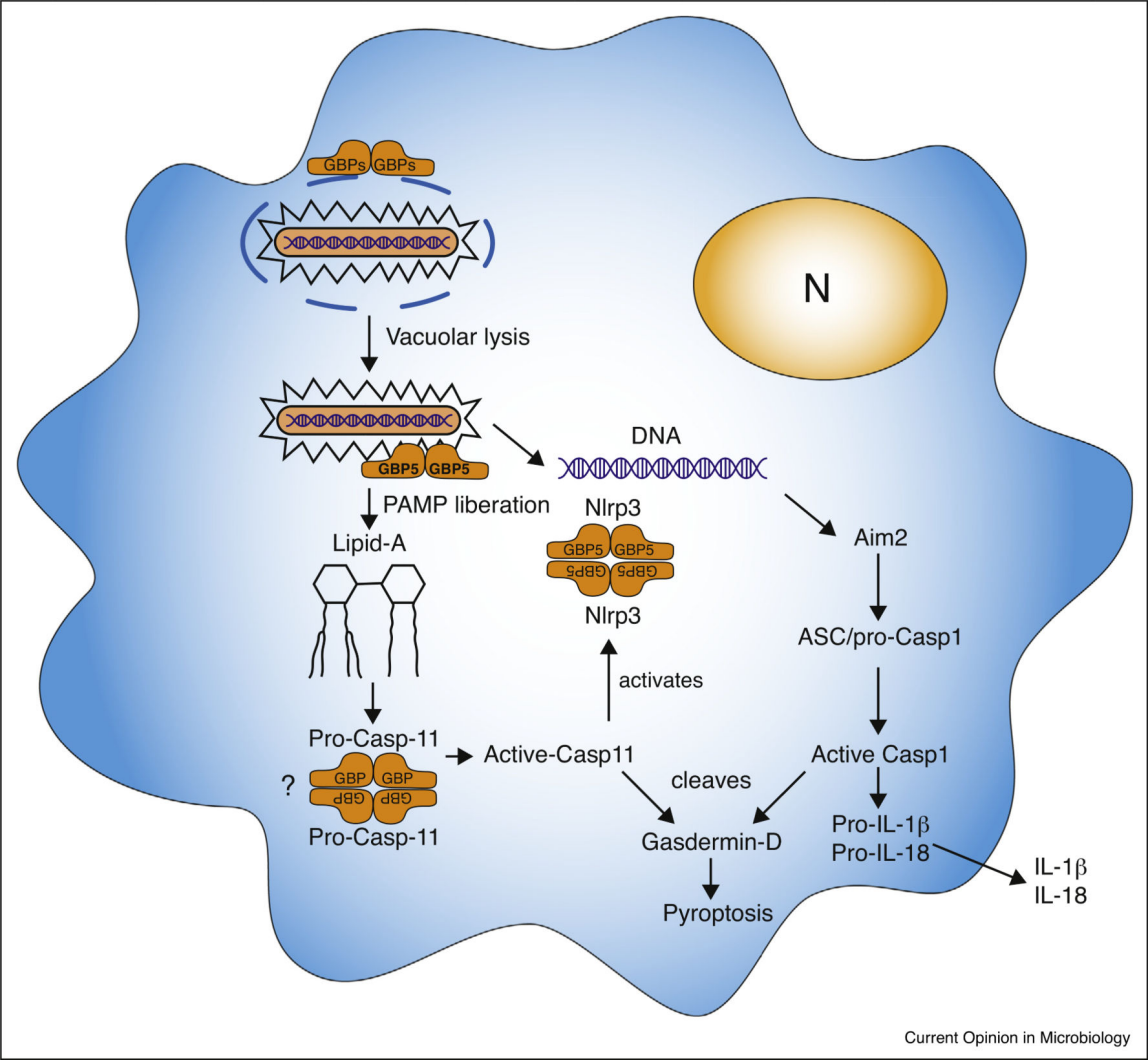
Inflammasome activation driven by GBPs. GBPs can mediate inflammasome activation by lysing the vacuole of pathogens and/or direct lysing cytosolic bacteria leading to the exposure of PAMPS such as LPS and DNA which can activate Casp11 and AIM2, respectively. Certain GBPs can also tetramerize and bind to Casp11 or Nlrp3 thereby lowering the threshold for their activation. For more details see main text.

GBPs can mediate the exposure of pathogen PAMPs to cytosolic PRR thereby activating the inflammasome and pyroptosis and two different mechanisms have been reported (Figure 3):

1. GBPs can direct the destruction of the vacuolar membrane of certain gram-negative bacteria thereby releasing Lipopolysaccharide (LPS) to the cytoplasm. The CARD domain of Casp11 (human CASP4/5) can directly bind to Lipid-A in aggregates of LPS resulting in Casp11 activation and Gasdermin-D-mediated host cell death. Casp11 also mediates the secretion of IL-1*β* and IL-18 via activation of the Nlrp3-Asc-Casp1 inflammasome through an unknown mechanism.
2. Instead of aiding the lysis of the vacuole, GBPs together with Irgb10 can localize to the bacterial cell membrane (and within the bacteria) of cytosolic bacteria (e.g. *Francisella* escaped from its vacuole). By vesiculating the bacterial membrane they mediate the release of LPS and DNA, PAMPs that can subsequently activate Casp11 and the AIM2 inflammasome [44,45].

GBP5 (or the GBP cluster on chr3, GBP^chr3^) is not needed for inflammasome activation upon *E. coli* LPS transfection, possibly because this results in high cytoplasmic LPS concentrations that might already be able to concentrate Casp11 sufficiently for its activation. In contrast, efficient L. *pneumophila* LPS detection is dependent on GBP^chr3^ and Casp11 [46] which might be because L.*pneumophila* lipid A has longer fatty acid chains compared to *E. coli* and *Salmonella* LPS.

It has also been shown that GBP5 is needed for Nlrp3 inflammasome activation of mouse BMDM and a human macrophage cell line stimulated with LPS and triggered by ATP or Nigericin or infection with *Salmonella* or *Listeria* [47]. Similarly, rapid inflammasome activation by *Chlamydia muridarum* was independent of vacuolar lysis by the GBPs, but rather involved GBP(chr3)-mediated Casp1/Casp11 Nlrp3 and AIM2 inflammasome activation [48^•^]. The GTPase domain of GBP5 was shown to bind to the pyrin domain of Nlrp3 and the multimerization of GBP5 was suggested to increase the local concentration of Casp1 bound to Nlrp3/Asc and thereby its activation [47]. Although other studies did not replicate the influence of GBP5 on the Nlrp3 inflammasome [49,50] it has been noted [7] that the genetic background of the region around the GBP5 deletion was 129 in the study by Shenoy et al. [47], while it was C57BL/6 in the Meunier study. C57BL/6 vs. A/J macrophage differences in Toxoplasmacidal activity maps to a region on Chr3 that contains the GBPs. C57BL/6, and a variety of other mouse strains, do not express GBP1 upon IFNγ induction, while A/J and 129 does [51], possibly explaining these mouse strain differences in Toxoplasmacidal activities [52].

If GBPs mediate inflammasome activation of parasites or viruses is currently unknown. The Nlrp3 and Nlrp1 inflammasome are important for *Toxoplasma* control in murine [53,54] and rat macrophages [55], respectively. Both Nlrp3 and Nlrp1 are important for *in vivo* murine control of *Toxoplasma* [54]. Neither the *Toxoplasma* molecules or cellular changes recognized by these inflammasomes nor the role of GBPs, if any, have been investigated. Rapid host cell death has been observed upon invasion of mouse and human IFNγ-stimulated fibroblasts by *Toxoplasma* [56,57] and *E. cuniculi* [58]. Death of IFNγ-stimulated fibroblasts upon infection with type II and III *Toxoplasma* strains was dependent on IRG-mediated destruction of the *Toxoplasma* vacuole membrane and did not resemble apoptosis nor was there cleavage of Caspase-1 or IL-1*β* [56]. However, pyroptosis can be activated without cleavage of Caspase-1 and therefore further experiments will have to investigate the exact mechanism of cell death.

## Conclusions

Much progress has been made toward the understanding of how the cell-autonomous defense to *Toxoplasma* is organized in IFNγ-stimulated murine cells. IRGs and GBPs as disruptors of the PVM are at the center of *Toxoplasma* counter-measures with the PV initially tagged by ubiquitin. This process is intimately driven by autophagy proteins. Exposed parasite material likely triggers host cell death, a response generally coordinated by GBPs in bacterial defense. It remains to be seen whether GBPs also mediate murine host cell death upon *Toxoplasma* infection.

The picture is less clear when considering the human host defense to *Toxoplasma*. Here, ubiquitin also plays a central role, but, the cell type drives the ultimate fate of the parasite - destruction versus growth restriction. Additionally, it remains to be seen whether the *Toxoplasma* restricting capacity of GBPs is at the PV or exerted from another location inside human cells. While tryptophan catabolism is an important IFNγ-mediated restriction mechanism in human cells, it is not clear how this pathway interacts with autophagy and GBPs. Most importantly, how *Toxoplasma* is sensed and subsequently restricted in human macrophages is not well understood. As technology advances, specifically with the advent of genome-wide host and parasite CRISPR screens [59,60] and stem cell technologies to generate non-transformed human cell types, our understanding of the human host defense in physiologically relevant cell systems to *Toxoplasma* could rapidly improve.

## Figures and Tables

**Table 1:** Summary of IRG/GBP mediated control of *Toxoplasma* in different murine and human cell lines. For references see main text.

	Mouse (MEF, macrophages, astrocytes)			Human epithelial A549	Human HAP1 (haploid fibroblastlike leukemia cell)
	IRGs	GBPs	*Toxoplasma* virulence factors	GBPs	GBPs
Recruited to the PV or *Toxoplasma*	Irgm2, Irgm3, Irga6, Irgb6, Irgd recruited to the PV	•Gbp1, 2, 3, 5, 7 recruited to PV •Gbp2 recruited to *Toxoplasma*	Combating GTPase recruitment: ROP18 (type I/II) ROP5 (type I/III)	GBP1 not recruited to PV	GBP1–5 recruited to 8% PVs
Ability to control *Toxoplasma*	Irgm1, Irgm3, Irga6, Irgd able to control *Toxoplasma* replication	•Gbps on chromosome 3 in bulk (1, 2, 3, 5, 7) control *Toxoplasma* replication •Gbp1 controls *Toxoplasma* replication	ROP16 (type I/III) ROP17 GRA7	GBP1 controls on of type II, but not type I	GBP1–5 do not control replication of type II
			Enhancing GTPase recruitment: GRA15 (type II)		
